# Coexistence of Lupus Nephritis, Ulcerative Colitis, and Communicating Hydrocephalus: A Report of a 21-Year-Old Male

**DOI:** 10.1155/2022/1079300

**Published:** 2022-02-04

**Authors:** Bushra Ali Khan, Nida Saleem, Danyal Hassan, Shabaz Kiani, Muhammad Haneef

**Affiliations:** Shifa International Hospital, H-8/4, Pitras Bukhari Road, Islamabad, Pakistan

## Abstract

Systemic lupus erythematosus (SLE) and ulcerative colitis (UC) are multisystem autoimmune disorders that rarely coexist. We report a case history of a 21-year-old male, presenting with bloody diarrhea and, later, diagnosed to have ulcerative colitis on colonic biopsy. There was clinically silent renal impairment leading to end-stage kidney disease requiring hemodialysis possibly secondary to ongoing lupus nephritis as suggested by positive lupus-specific antibodies' detection. Besides this, the diagnosis of lupus associated with early communicating hydrocephalus was made on CT brain findings which clinically responded well to the initiation of immunosuppressive therapy. It is imperative to keep in mind the remote possibility of ulcerative colitis in an SLE patient with gastrointestinal (GI) manifestations. Communicating hydrocephalus is a rare neurological manifestation of SLE leading to seizures and can respond well to the initiation of steroids and immunosuppressants. Therefore, a trial of immunosuppressant medications must be given even in a patient with end-stage renal disease (ESRD) to halter extra renal rare lupus manifestations.

## 1. Introduction

Inflammatory bowel disease is a recurrent inflammatory autoimmune disorder involving gastrointestinal mucosa, and it includes Crohn's disease and ulcerative colitis [1]. SLE is an immune complex-mediated disorder common in women of reproductive age group. Although it has been known that many autoimmune disorders coexist, yet, coexistence of SLE and ulcerative colitis has rarely been reported. The reported prevalence of these two disorders is only 0.4% [1, 2]. These two autoimmune disorders share certain common features [3]. In SLE, gastrointestinal manifestations are very common which might be secondary to the disease itself, superimposed infections, or side effects of immunosuppressant medications. SLE can lead to gastrointestinal (GI) vasculitis, peritonitis, pancreatitis, mesenteric ischemia, peptic ulcer disease, acalculous cholecystitis, pseudo-obstruction, and lupus enteritis [4]. Similarly, there are certain manifestations of ulcerative colitis that are similar to SLE. These include oral ulcers and arthritis [3], thus making diagnosis of these coexistent autoimmune disorders extremely difficult.

Although the reported prevalence of neuropsychiatric manifestations of SLE is 50% which include stroke, meningitis, movement disorders, myelopathy, and psychiatric features, the occurrence of communicating hydrocephalus is extremely rare. So far, only a few case series and reports are available [5]. It has been seen that, in patients with coexistent SLE and ulcerative colitis, the overall course of SLE is benign with no marked renal impairment [2] and neurological involvement [3]. However, we report a rare case of coexistent SLE with clinical presentation of ulcerative colitis in a 21-year-old male, requiring permanent hemodialysis secondary to possible lupus nephritis and also who developed lupus-induced nonobstructive hydrocephalus clinically responding well to immunosuppressive therapy.

## 2. Case

A 21-year-old male, an average student, with no previous comorbidities, was on hearing aid due to hearing impairment noted after several months of a noneventful birth. He was born at 38 weeks of gestation with average birth parameters including weight and height and no immediate developmental abnormality. No family history of hearing loss and chronic kidney disease or ESRD was found among family members. During this admission, he presented with a complaint of bloody diarrhea for one month. Diarrhea was watery in consistency, containing clots of blood, and had a frequency of 7-8 times per day. He initially went to a local hospital, and colonoscopy and colonic biopsy were done, approximately after 20 days of the onset of symptoms. The colonoscopy report showed inflamed and edematous colonic mucosa with multiple bleeding ulcers. The histopathological report showed evidence of focal active colitis with crypt abscess. In order to manage him in lines of inflammatory bowel disease, he was started on tablet mesalazine, loperamide, pantoprazole, rifaximin, and mebeverine. However, he remained noncompliant with medications, and his bloody diarrhea persisted.

Later, he was brought to the Shifa International Hospital emergency department (ER) in a severely dehydrated state with altered mental status. As per the family, there were complaints of generalized weakness, drowsiness, reduced appetite, hiccups, and decrease in the urine output for 1 day. He also had off and on pain in both knee joints for the last 45 days. However, there were no complaints of vomiting, abdominal pain, and fever. He was semioriented and semiresponsive. His vitals at the time of presentation showed a blood pressure of 110/60 mmHg and pulse of 110/min, and he was afebrile. He was clinically pale with sunken eyes and reduced skin turgor. There were no skin rashes, oral ulcers, and enlarged lymph nodes. His systemic examination was unremarkable. He was catheterized immediately, and 500 ml bolus of bicarbonate-based IV fluid was given. His laboratory investigations were sent, which are summarized in Table 1.

**Figure 1:**
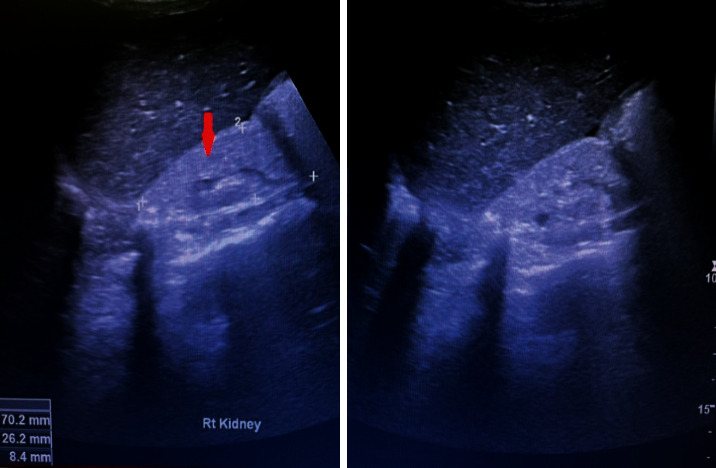
Grade III echogenic right kidney, 70.2 × 26.2 × 8.4 mm in dimensions (red arrow).

Ultrasound of the abdomen was performed in the ER which showed bilateral small echogenic kidneys with grade III renal parenchymal echogenicity as shown in Figure 1.

Given the degree of chronicity and severe metabolic and electrolyte abnormalities, urgent hemodialysis was initiated in the ER via the left femoral temporary dialysis catheter. First session was performed for 2 hours due to marked hyponatremia and uremia with one pack of blood transfusion. He was started on intravenous meropenem and vancomycin given the raised inflammatory and infectious markers.

After a total of 3 sessions of hemodialysis, his conscious level improved. His BUN improved to 77 mg/dL and sodium to 132 mEq/L over 72 hours. Creatinine improved to 4.58 mg/dL and bicarbonate to 22 mEq/L.

In order to identify the exact cause of his renal impairment, his autoimmune workup was sent, which is shown in Table 2.

Table 2 shows low c3 levels and positive ANA, anti-double-stranded (ds) DNA, anti-Golgi apparatus antibodies, and anti-Smith antibodies consistent with the diagnosis of SLE.

Regarding systemic lupus erythematosus (SLE) further management, the rheumatology and gastroenterology departments were taken on board. The patient was initiated on intravenous methylprednisolone pulse therapy which was given at a dose of 500 mg once daily for 5 days, along with mycophenolate mofetil (MMF) at a dose of 1 g twice daily and hydroxychloroquine (HCQ) 200 mg once daily.

A repeat colonoscopy with colonic biopsy was performed. On colonoscopy, multiple ulcers were present throughout the colon, more marked in the terminal ileum leading to narrowing and deformation of the ileocecal valve. Beside pulse methylprednisolone, the patient was prescribed per oral tablet mesalazine 1 g thrice daily.

**Figure 2:**
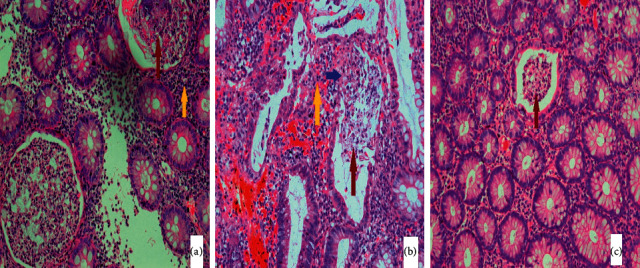
(40x): hematoxylin-and-eosin-stained light microscopic images (a–c). Evidence of crypt abscesses (maroon arrow), marked lymphocytic infiltration of the lamina propria (yellow arrow), and colonic mucosal ulceration with mucin depletion.

The histopathology report of the terminal ileum and colonic biopsy showed evidence of moderate chronic active colitis consistent with ulcerative colitis, along with ulceration of colonic mucosa with slough formation and mucin depletion. Besides this, cryptitis and crypt abscesses were also seen. There was evidence of mixed inflammatory infiltrates composed of lymphocytes, plasma cells, neutrophils, and eosinophils in the lamina propria. These findings are shown in Figure 2.

Later on, during the hospital stay, the patient had one episode of generalized tonic clonic seizure associated with frothing from the mouth and uprolling of eyes. While being managed in the Neurology ICU, he suffered two more episodes of seizures, which were settled with intravenous diazepam. Neurology team was taken on board, and levetiracetam was given intravenously at a loading dose and then continued at a maintenance dose. EEG and plain CT scan brain were performed.

**Figure 3:**
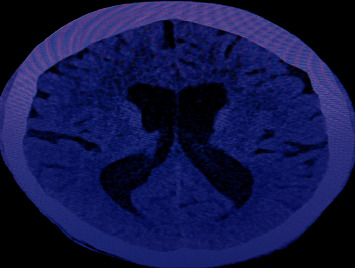
Dilated lateral ventricle consistent with hydrocephalus.

CT scan brain without contrast showed evidence of early communicating hydrocephalus. There was dilatation of lateral, third, and fourth ventricles with rounding of frontal horns and empty sella. However, posterior fossa structures including the cerebellum and CP angle cisterns were unremarkable. CT image of the patient is shown in Figure 3.

At that time, his serum sodium was 125 mEq/L, serum magnesium was 1.6 mg/dL, and serum calcium level was also 7.3 mg/dL. Electrolyte imbalance was corrected in the ICU setting, and intravenous mannitol was also given. Lumbar puncture was also advised by the neurological team to rule out the possibility of meningitis. However, the family refused due to financial issues. As the patient's condition stabilized, he was transferred back to the ward after 24–48 hours. His temporary dialysis catheter was then removed, and later on, the right internal jugular tunneled catheter was inserted.

With the initiation of immunosuppressive therapy, antiseizure medications, and correction of electrolyte imbalance and uremia, the patient's level of consciousness improved to his baseline normal. Frequency of diarrhea also reduced, and seizures subsided on per oral levetiracetam; therefore, he got discharged on MMF, hydroxychloroquine (HCQ), levetiracetam, calcium carbonate, calcium acetate, erythropoietin, mesalazine, pantoprazole, and prednisolone.

After two weeks, on follow-up visit, he had gained weight as his appetite improved, and his diarrhea settled, with no seizure activity reported with baseline normal mental status. All his laboratory parameters including CRP and procalcitonin improved. The patient was continued on the tapering dose of steroids, MMF, HCQ, levetiracetam, and erythropoietin and was instructed to continue regular hemodialysis sessions.

Unfortunately, a repeat CT brain was not done due to noncompliance and reluctance on part of the family due to financial strains.

## 3. Discussion

In summary, we report the first case of lupus nephritis leading to ESRD and communicating hydrocephalus with coexistent ulcerative colitis that has responded well to initiation of steroids and immunosuppressive medications.

It has been suggested as per findings of previously reported cases that SLE precedes ulcerative colitis by several years and usually remains clinically silent with less frequent arthritis, serositis, and other systemic manifestations [2]. Our case history supports this preposition as our patient has clinically silent SLE leading to possible lupus-induced renal impairment, which, later, was diagnosed on a detailed autoimmune workup suggestive of SLE. However, biopsy-proven confirmation of lupus nephritis was not possible due to bilateral shrunken grade-3 echogenic kidneys.

As per previously studied data, it has been suggested that SLE usually runs an indolent course if it coexists with ulcerative colitis; so far, no reported data of severe renal and neurological involvement were obtained [2]. In one systemic review of 9 diagnosed cases of coexistent UC and SLE, not even a single neurological manifestation and dialysis-dependent marked renal impairment was reported [3]. However, as seen from our patient, we can suggest that coexistent UC and SLE can lead to marked renal impairment and even neurological involvement.

Regarding the autoimmune profile, it has been suggested that anti-dsDNA antibodies are positive in 100% of previously reported cases [2, 3], and anti-LA antibodies [6] are also positive in the majority of cases. The finding of anti-dsDNA positivity seen in our patient is consistent with this preposition.

It is well known that the use of certain drugs for the treatment of ulcerative colitis such as sulfasalazine can lead to drug-induced lupus [1, 7]. Furthermore, the use of infliximab for ulcerative colitis has also been associated with the development of hydrocephalus [8]. However, our patient has already developed ESRD prior to mesalazine use, and infliximab has not been prescribed to our patient, thus ruling out these two possibilities.

In SLE, the possible causes of hydrocephalus include superimposed intracranial opportunistic infection, cerebral phlebitis, antiphospholipid antibody (APLA) [9] mediated cerebral venous thrombosis, aseptic meningitis, SLE-induced vasculitis with subarachnoid hemorrhage [5], and postinflammatory aqueductal stenosis [10]. Moreover, the pathological mechanism of SLE-induced hydrocephalus can either be due to direct neuronal and vascular damage or immune complex-mediated and hypercoagulability-induced damage to arachnoid villi, leading to the obstruction of CSF outflow. In our case, the second mechanism is likely a contributing factor in the development of communicating hydrocephalus. However, due to refusal of lumbar puncture and CSF analysis, the possibility of infection-induced nonobstructive hydrocephalus cannot be ruled out. In our patient, the negative APLA antibody workup and coagulation profile are suggestive of non-APLA-associated communicating hydrocephalus.

Regarding the treatment of autoimmune disorder-induced hydrocephalus, several options have been proposed. These include medical management with or without ventriculoperitoneal (VP) shunting [5]. Steroids have been considered as the cornerstone of medical management in autoimmune disorder-induced hydrocephalus. However, the reported outcome has been much better with VP shunting as compared to medical management [5]. As our patient had early-onset communicating hydrocephalus and seizures with coexistent electrolyte abnormalities, therefore, we had given a trial of medical management with simultaneous correction of electrolyte imbalances. Unfortunately, a repeat CT brain was not done due to noncompliance and reluctance on part of the family due to financial strains. Hence, we cannot for sure report an improvement in hydrocephalus, though the patient's clinical improvement may suggest an improvement in the neurological involvement of SLE and hydrocephalus.

The major limitation of this case report is the lack of histopathological confirmation of lupus nephritis due to evidence of bilateral grade-3 echogenic, shrunken kidneys which can predispose to postprocedure complications. However, the presence of positivity of ANA, anti-dsDNA, anti-Smith, and anti-Golgi apparatus antibodies along with hypocomplementemia, proteinuria, and hematuria is highly suggestive of the likely diagnosis of lupus nephritis.

## 4. Conclusion

From this case description, it can be concluded that SLE and ulcerative colitis can coexist, though rarely as suggested by the previous case reports. Therefore, in an SLE patient with GI manifestation, the remote possibility of UC must be kept in mind. As reported previously, SLE usually runs an indolent course, though, in our case, we have shown that SLE can present with severe symptoms including renal insufficiency and ESRD. Although hydrocephalus is a rare manifestation of SLE, depending on the patient's symptoms and severity of ventricular dilatation, decision must be made promptly whether to manage it medically alone or with surgical intervention. However, in less severe cases, a trial of steroids and immunosuppressant medications can be considered alone.

## Figures and Tables

**Table 1:** Postadmission laboratory investigations.

Hemoglobin (g/dL)	7.1	Serum sodium (mEq/L)	119
Total leucocyte count (cells/cmm)	9490	Serum bicarbonate (mEq/L)	9
Platelet count (cells/uL)	201000	Serum potassium (mEq/L)	5.2
Folic acid	3.1	Serum chloride (mEq/L)	97
Vitamin B12 levels	189	Serum creatinine (mg/dL)	17.2
TSAT	32.5%	BUN (mg/dL)	140
Coagulation profile	Negative	Serum calcium (mg/dL)	7.3
ESR (mm/hour)	140	Serum phosphorous (mg/dL)	4.2
CRP (mg/L)	95.38	Serum albumin (mg/dL)	1.7
Procalcitonin	19.22	Urine R/E	Protein: +, blood: +++, RBCs: 20–25/HPF, WBCs: 1-2/HPF
Stool R/E	No cyst, ovum, or worm	Spot urine protein-to-creatinine ratio	2.2 g/g
Stool for CMV	Negative	Blood C/S	Negative
QuantiFERON-TB	Negative	Urine C/S	Negative
Serum chloride (mEq/L)	97		

ESR: erythrocyte sedimentation rate; R/E: routine examination; RBCs: red blood cells; WBCs: white blood cells; CMV: cytomegalovirus; BUN: blood urea nitrogen.

**Table 2:** Autoimmune workup.

ANA	Positive (1 : 80)	Anti-dsDNA antibodies (IU/mL)	Positive (55)
C3	0.67	Anti-Smith antibodies (U/mL)	Positive (37)
c-ANCA	Negative	Antihistone antibodies	Negative
p-ANCA	Negative	Hepatitis B S Ag	Negative
Anti-GBM	Negative	Hepatitis C antibodies	Negative
Anticardiolipin antibodies	Negative	Antilupus anticoagulant antibodies	Negative
Anti-beta-2 glycoprotein antibodies	Negative	Anti-Golgi apparatus antibodies	Positive (1 : 160)

c3: complement-3; p-ANCA: perinuclear antineutrophilic cytoplasmic antibodies; c-ANCA: circulating antineutrophilic cytoplasmic antibodies; ANA: antinuclear antibody; S Ag: surface antigen; ds: double-stranded; anti-GBM: antiglomerular basement membrane antibodies.

## Data Availability

The data that support conclusions can be accessed through references mentioned in the discussion.
